# Ciprofloxacin-resistant *A. hydrophila* complicating leech therapy in free flap surgery

**DOI:** 10.1016/j.jpra.2025.04.003

**Published:** 2025-04-11

**Authors:** Mai Nishijo, Calver Pang, Keith Anderson, Charles Yuen Yung Loh

**Affiliations:** a Department of Plastic Surgery, Cambridge University Hospital NHS Foundation Trust, Hills Road, Cambridge, United Kingdom; b Department of Surgical Biotechnology, Division of Surgery and Interventional Science, Faculty of Medical Sciences, University College London, Gower Street, London, United Kingdom

*Dear Sir,*

Medicinal leeches (*Hirudo medicinalis*) play a critical role in managing venous congestion following free flap reconstruction. Their efficacy is attributed to the vasodilatory and anticoagulant properties of their saliva, which facilitate venous drainage. However, the use of leech therapy carries a well-documented risk of secondary infection due to *Aeromonas hydrophila*, a bacterium residing in the leech gut. To mitigate this risk, prophylactic antibiotics such as ciprofloxacin are commonly prescribed.

Previous literature^1^, ^2^, ^3^ has highlighted cases of ciprofloxacin-resistant *Aeromonas hydrophila*, raising concerns about the effectiveness of this prophylactic strategy. Here, we present a case of a patient treated with leech therapy for venous congestion in a fibular free flap, whose postoperative course was complicated by cellulitis due to ciprofloxacin-resistant *A. hydrophila*.

A 63-year-old male sustained a complex open forearm fracture with extensive segmental loss of the radius following a road traffic accident. Following primary debridement, the defect was reconstructed with a flow-through fibular free flap to the radius and ORIF of the ulnar shaft. Few hours postoperatively, venous congestion developed at the proximal end of the flap, prompting initiation of leech therapy. Prophylactic ciprofloxacin, at a dose of 500mg twice daily orally, alongside co-amoxiclav for the open fracture, was administered for a 48-hour course of leech therapy.

Twelve days later, the patient developed erythema and sloughy discharge from the proximal end of the flap (Figure 1). Empiric antibiotic therapy with vancomycin and piperacillin-tazobactam was initiated as per bone infection team, and the patient underwent surgical debridement and washout. Intraoperative findings confirmed superficial infection without deeper collections. Tissue culture sensitivities identified *A. hydrophila* resistant to ciprofloxacin and co-trimoxazole but sensitive to aztreonam, ceftazidime, and meropenem. The patient completed a 7-day course of vancomycin and ceftazidime, with subsequent resolution of cellulitis (Figure 2). The specific batch of leeches was flagged to the hospital pharmacy for Yellow Card reporting.

**Figure 1:**
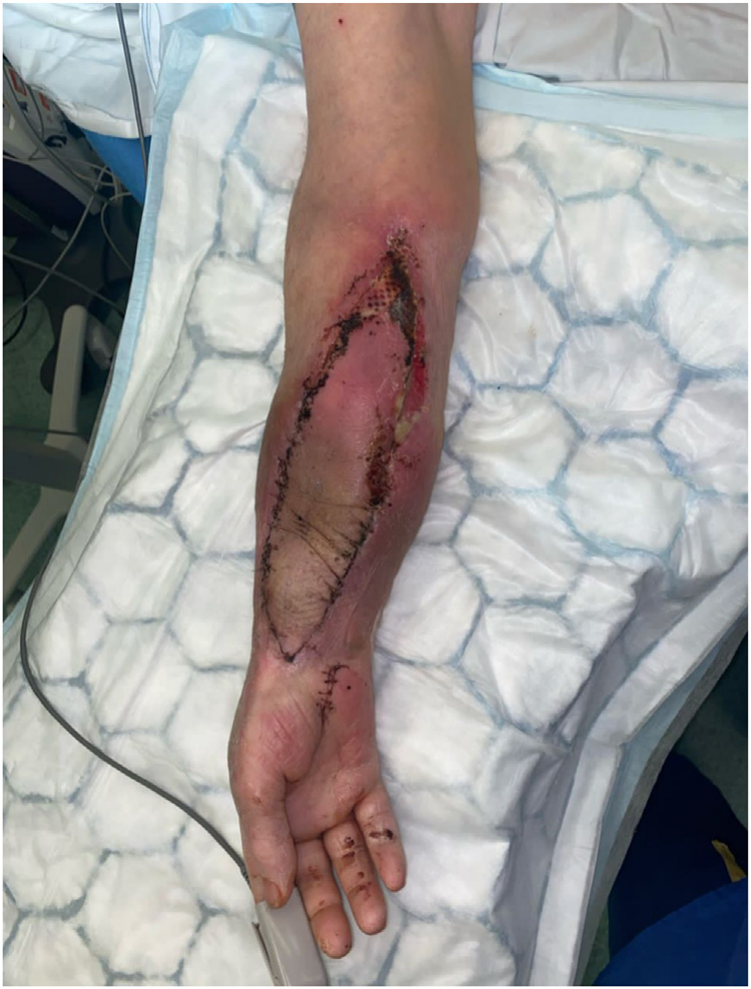
Image of cellulitis in a fibular free flap due to ciprofloxacin-resistant Aeromonas hydrophila infection.

**Figure 2:**
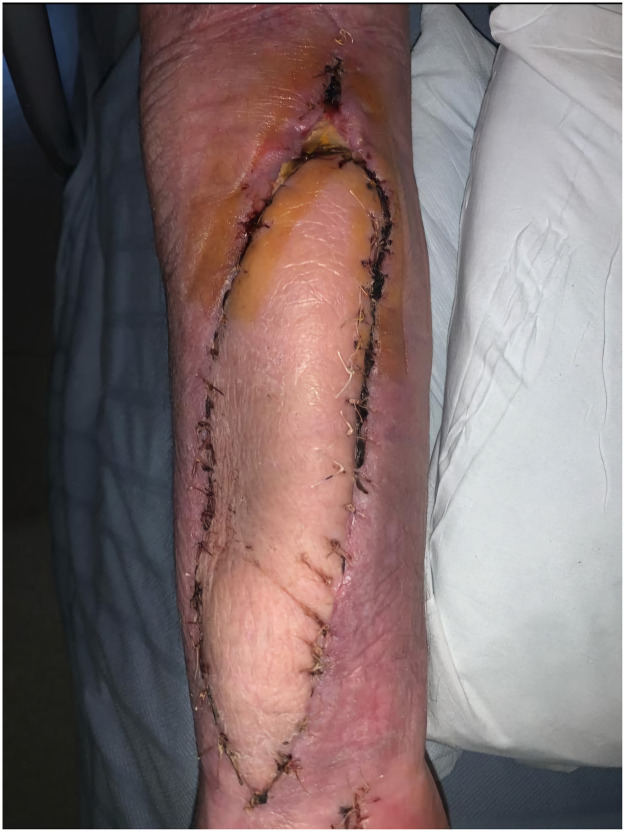
Image showing resolution of cellulitis in a fibular free flap following targeted antibiotic therapy.

Prompt re-exploration for venous congestion of the free flap was not undertaken, as the main skin paddle area remained well perfused and the congested section was outside the primary vascular territory (angiosome). However, in cases of venous congestion, early re-exploration is recommended, as it offers the best chance of restoring venous outflow and ensuring flap survival. Nonetheless, leech therapy remains a valuable non-surgical option in patients with high surgical risks, such as in elderly individuals or those with multiple comorbidities, or in cases where intraoperative anastomosis was particularly challenging.

This case, along with previous reports, underscores the growing clinical relevance of ciprofloxacin-resistant *A. hydrophila* associated with leech therapy. Our findings highlight the need for increased awareness of this resistance and consideration of early debridement to enable timely management of arising complications. Additionally, this suggests the necessity of reassessing current prophylactic protocols and considering alternative or adjunctive antibiotic regimens to mitigate this emerging risk. In considering alternative prophylactic treatments, it's important to recognize that *A. hydrophila* can produce beta-lactamases. Co-trimoxazole, another potential prophylactic agent,^4^ may also be subject to resistance, as demonstrated in our patient. Given the potential risks associated with resistant strains, further research and consensus on prophylactic strategies are crucial, with early consideration of surgical intervention, to ensure the continued success of leech therapy in reconstructive surgery.

## Declaration of competing interest

None.
